# Society for Cardiovascular Magnetic Resonance (SCMR) recommended CMR protocols for scanning patients with active or convalescent phase COVID-19 infection

**DOI:** 10.1186/s12968-020-00656-6

**Published:** 2020-09-03

**Authors:** Sebastian Kelle, Chiara Bucciarelli-Ducci, Robert M. Judd, Raymond Y. Kwong, Orlando Simonetti, Sven Plein, Francesca Raimondi, Jonathan W. Weinsaft, Timothy C. Wong, James Carr

**Affiliations:** 1 DZHK (German Centre for Cardiovascular Research), Berlin, Germany; 2 German Heart Institute Berlin and Charité University Medicine Berlin, Augustenburger Platz 1, 13353 Berlin, Germany; 3 Bristol Heart Institute, Bristol NIHR Biomedical Research Centre, University Hospitals Bristol and University of Bristol, Bristol, UK; 4 Department of Cardiology, Duke University, Durham, North Carolina USA; 5 Cardiac Magnetic Resonance Imaging, Cardiovascular Division, Department of Medicine, Brigham and Women’s Hospital, Harvard Medical School, 75 Francis Street, Boston, MA 02115 USA; 6 Departments of Internal Medicine and Radiology, The Ohio State University, Columbus, OH USA; 7 Leeds Institute for Genetics Health and Therapeutics & Leeds Multidisciplinary Cardiovascular Research Centre, University of Leeds, Leeds, UK; 8 Centre de référence “Malformations Cardiaques Congénitales Complexes – M3C” Service de Cardiologie Pédiatrique Hôpital Necker-Enfants Malades, Université Sorbonne Paris Cité, Paris, France; 9 Department of Medicine, Weill Cornell Medicine, New York, NY USA; 10 Heart and Vascular Institute, University of Pittsburgh Medical Center, Pittsburgh, PA USA; 11 Department of Radiology, Feinberg School of Medicine, Northwestern University, Chicago, IL USA

**Keywords:** Recommendations, CMR, COVID-19, Protocol, Indication, SCMR, SARS-CoV-2, Cardiac MRI, Pandemic, Heart

## Abstract

The aim of this document is to provide specific recommendations on the use of cardiovascular magnetic resonance (CMR) protocols in the era of the COVID-19 pandemic. In patients without COVID-19, standard CMR protocols should be used based on clinical indication as usual. Protocols used in patients who have known / suspected active COVID-19 or post COVID-19 should be performed based on the specific clinical question with an emphasis on cardiac function and myocardial tissue characterization. Short and dedicated protocols are recommended.

## Purpose

Society for Cardiovascular Magnetic Resonance (SCMR) guidance for the practice of cardiovascular magnetic resonance (CMR) during the COVID-19 pandemic have recently been published [1]. The aim of this document is to provide more specific recommendations on the CMR protocols for scanning patients with known or suspected severe acute respiratory syndrome coronavirus 2 (SARS-CoV-19) infection or those recovering from the disease. These recommendations are not meant to be restrictive but rather to serve as a general framework. They also serve as the basis for registries and clinical studies in patients with SARS-CoV-2 infection, to ensure consistency between local, national and international efforts. All recommendations will be updated continuously and provided online in the SCMR’s coronavirus disease 2019 (COVID-19) Preparedness Toolkit https://scmr.org/page/COVID19.

## Common CMR-indications in patients with COVID-19

The effects of COVID-19 on cardiac structure, function, and tissue properties are largely uncertain. First reports are indicating potential myocardial injury or involvement in the acute phase of COVID-19 and post infection [2–9]. CMR is the non-invasive imaging method of choice for structural and functional evaluation of the heart and has unique tissue characterization abilities and will therefore play a key role in many patients recovering from COVID-19 and cardiac involvement [10–12]. Adults and children appear to have different presentations with some reports of children being affected by a hyperinflammatory syndrome with acute myocarditis or Kawasaki-like clinical presentation [13, 14]. Based on recent literature, the following are the most likely indications for CMR in patients with active or convalescent phase of COVID-19:

### Expected CMR-indications in patients within active or convalescent phase of COVID-19

**Table:** 

Adults: - Left- and right ventricular dysfunction (heart failure) - Myocarditis (including systemic inflammatory disease, cardiotoxicity) - Pericarditis - Myocardial infarction with non-obstructive coronary arteries (MINOCA) - Chest pain (chronic coronary syndrome) - Acute myocardial infarction - Stress induced cardiomyopathy (Takotsubo) - Ventricular arrythmia, resuscitated cardiac arrest - Pulmonary hypertension - Vasculitis Children - Left- and/or right ventricular dysfunction (heart failure) - Hyperinflammatory syndrome or Kawasaki-like features - Acute vasculitis - Cardiogenic shock	

### CMR protocols

In many clinical scenarios, CMR protocols in the context of COVID-19 infection will need to be short and adapted to the breath-hold ability of patients. Short protocols also help to minimize exposure risks for healthcare personal and patients (Table). Whenever possible, CMR studies should be postponed until patients are no longer contagious so as to avoid exposure to scan personnel. Recently, specific recommendations how to perform CMR in the current COVID-19 pandemic have been published by the SCMR [1] and European Association of Cardiovascular Imaging (EACVI) [15]. Acquisition and reporting should follow general SCMR guidance [16, 17]. If contrast administration is planned, check of kidney function is recommended in COVID-19 patients, however not mandatory particularly with appropriate (macrocyclic) contrast agents. Wherever possible, a COVID-19 dedicated CMR scanner should be used for scanning patients with active infection.

### Recommended CMR-protocols in adult patients with active/post COVID-19

**Table:** 

**Recommended CMR-sequences**	**Answering most clinical questions**
Survey	Minimum
Cine sequences (SAx full coverage, LAX: 4Ch; 2Ch; 3Ch)	Minimum
T2- weighted sequences (STIR) (myocardium/pericardium)	Desirable
Parametric Mapping (T1-native, T2, T1-post (calculate ECV)	Desirable
Acquisition based myocardial strain (Tagging, DENSE, fSENC)	Optional
Stress perfusion (vasodilator)	Optional
Late gadolinium enhancement (LGE)	Minimum (SAx) Desirable (long axis)
2D-flow (aorta & pulmonary arteries)	Desirable
4D-flow	Optional
Angiography (pulmonary vessels)	Optional
Lung imaging	Optional

2Ch = two chamber; 3Ch = three chamber; 4Ch = four chamber; DENSE = displacement encoding with stimulated echoes; ECV = extracellular volume fraction; LGE = late gadolinium enhancement; SAx = short axis; SENC = strain encoding; STIR = short tau inversion recovery

### Recommended CMR-protocols in children with active/post COVID-19

**Table:** 

**Recommended CMR-sequences**	**Answering most clinical questions**
Survey	Minimum
Cine sequences (SAX full coverage, LAX: 4-CH; 2-CH; 3-CH)	Minimum
T2- weighted sequences (STIR) (myocardium/pericardium)	Minimum
Parametric Mapping (T1-native, T2, T1-post (calculate ECV)	Desirable
Acquisition based myocardial strain (Tagging, DENSE, fSENC)	Desirable
Late gadolinium enhancement (LGE)	Minimum (SAX) Desirable (long axis)
2D-flow (aorta & pulmonary arteries)	Desirable
4D-flow	Optional
Angiography (pulmonary vessels)	Optional
3D sequence for coronary artery anatomy	Desiderable
Lung imaging (T2 weighted Fat-suppressed sequences with motion correction (free breathing with respiratory triggered) for whole thorax in axial plane)	Desiderable

In patients with active COVID-19 or for those with poor functional status, a short protocol (10–15 min) including a minimum data set of cardiac function and focal myocardial damage (late gadolinium enhancement (LGE)- minimum single shot in short axis (SAx)-orientation) is recommended. In children with active/post COVID-19 a 3D sequence for coronary artery imaging to detect eventual coronary dilation/ectasia in proximal or mean segments of the coronary artery tree is recommended. Data acquisition should be as short as possible by using undersampling methods and or reductions in spatial or temporal resolutions without loss of diagnostic image quality. Use of free-breathing and real-time acquisition methods is encouraged. If the patient’s functional status allows a scan time of around 30 min, components indicated as “Desirable” in the table should be added to allow advanced tissue characterization and potential assessment of myocardial ischemia. Patients with acute COVID-19 infection often have respiratory compromise – in which context risks of vasodilator (regadenoson/adenosine) stress increased: Utility of pharmacolologic stress should be considered on a patient specific basis, considering concomitant risks in the context of active COVID-19 infection. Optional components of the protocol can be added according to the clinical question and patient condition. In addition, for parametric mapping and tissue characterization, hematocrit should be noted at the time of the CMR exam. Documentation of blood-pressure and heart rate at the time of the CMR scan is recommended.

### Scan interpretation and reporting

When interpreting CMR-exams, SCMR reporting guidelines should be followed [16, 17]. Based on literature published to date, potential findings in COVID-19 patients include acute and chronic myocarditis, pericarditis, left and right heart failure, acute coronary syndromes, myocardial infarction with normal coronary arteries, Kawasaki-like coronary ectasia (expecially in children) and Takotsubo cardiomyopathy. In addition, there may be a high incidence of non-cardiac findings, specifically pulmonary infiltrates or peripheral pulmonary vascular anomalies, in patients with COVID-19. If additional (or still persistent) pulmonary abnormalities are present at CMR-exams, further evaluation should be recommended.

In order to improve understanding of the cardiovascular effects of SARS-CoV-19 infection, SCMR is setting up a global CMR registry with adult (CMR COVID-19) and pediatric sections (CARDOVID study). Clnicians/scientists/researchers involved in reporting CMR scans in COVID-19 patients are encouraged to contribute to this effort. More information at www.scmr.org. Individual cases can be published in the SCMR COVID Case repository at www.scmr.org.

## Conclusion

According to the clinical indication, standard or rapid protocols should be used for COVID-19 patients. Especially short and dedicated CMR examinations that focus on the evaluation of cardiac morphology and function, as well as myocardial tissue characterization are recommended.

## Data Availability

‘Not applicable’

## Abbreviations

2Ch: Two-chamber
3Ch: Three-chamber
4Ch: Four-chamber
CMR: Cardiovascular magnetic resonance
COVID-19: Coronavirus disease 2019
DENSE: Displacement encoding with stimulated echoes
EACVI: European Association of Cardiovascular Imaging
LGE: Late gadolinium enhancement
MINOCA: Myocardial infarction with non-obstructed coronary arteries
SARS-CoV-2: Severe acute respiratory syndrome coronavirus 2
Sax: Short axis
SCMR: Society for Cardiovascular Magnetic Resonance
fSENC: (fast) Strain encoding
STIR: Short tau inversion recovery
